# Second primary common bile duct neuroendocrine tumor after 10 years in a patient with rectal adenocarcinoma: A case report

**DOI:** 10.22088/cjim.15.2.360

**Published:** 2024

**Authors:** Saba Ebrahimian, Sakineh Soleimani Varaki, Farid Nasr Esfahani

**Affiliations:** 1 Department of Surgery, Babol University of Medical Sciences, Babol, Iran; 2 Clinical Oncologist (Radiotherapy & Oncology), Babol University of Medical Sciences, Babol. Iran; 3 Department of Surgery, Yale New Haven Hospital, Yale University

**Keywords:** Neuroendocrine tumors, Ampullary neuroendocrine cancer, Second primary tumor

## Abstract

**Background:**

With the improvement of cancer treatment methods and increased life expectancy of patients, the prevalence of second primary cancers has gradually increased.

**Case Presentation:**

In the present study, the case was a 58-year-old man diagnosed with rectal adenocarcinoma and underwent chemotherapy and neoadjuvant radiotherapy 10 years ago. After 5 years, he underwent a lobectomy due to lung metastasis. At the research time, he presented with itching; in ERCP, a 16-millimeter hypoechoic lesion was detected, and an FNA biopsy was performed, indicating malignancy. The patient underwent Whipple surgery, and pathology revealed a well-differentiated neuroendocrine tumor. He was discharged in good general condition.

**Conclusion:**

This article emphasized the necessity of early detection and diagnosis of second primary cancer, as well as acting as if it was primary cancer to treat.

In cancer patients, the likelihood of a second primary cancer is generally overlooked. Understanding the clinical characteristics of second cancer in patients with a history of colorectal carcinoma is critical for early diagnosis and treatment (1). Most neuroendocrine tumors are associated with synchronous or metachronous second cancers (2). A few examples of synchronous colorectal cancer and gastrointestinal neuroendocrine tumors have been documented in the literature (3-5). However, the formation of a primary pancreatic neuroendocrine tumor following the complete resolution of colorectal cancer has not previously been reported. This study reported a patient with rectal adenocarcinoma who developed a neuroendocrine pancreatic tumor as a second primary cancer after surgical and chemotherapeutic treatments for lung metastatic colorectal malignancy.

## Case Presentation

**Figure 1:**
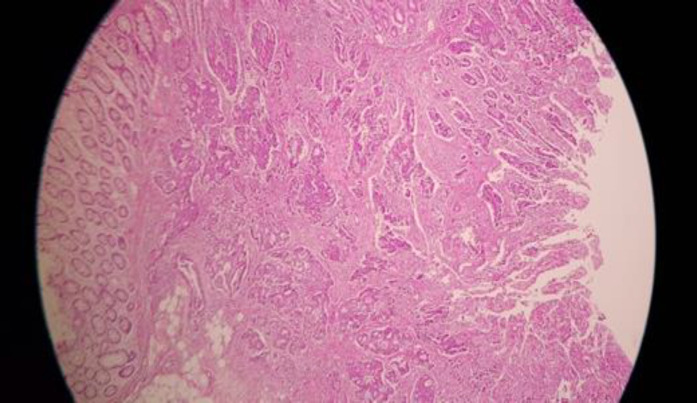
Rectal adenocarcinoma

**Figure 2:**
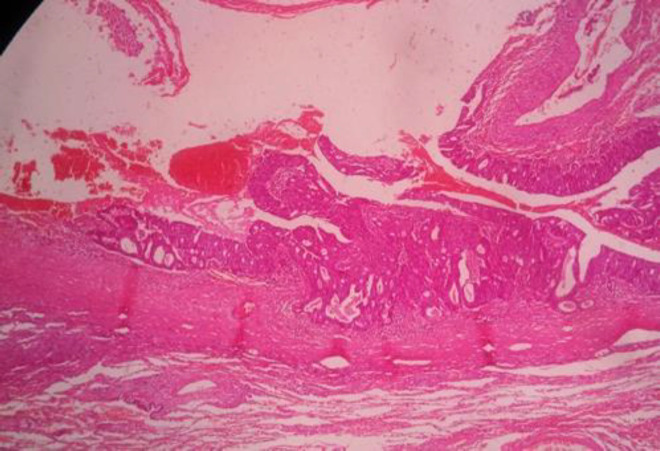
Lung metastasis

A 58-year-old man presented with icterus and pruritus 5 years after being diagnosed with colorectal metastatic lung diseases. He had a history of a ten-centimeter ulcerative mass above the anal verge, which had been diagnosed as rectal adenocarcinoma stage two 10 years before (figure 1). This patient received neoadjuvant chemotherapy with Irinotecan, 5Florouracil (8 courses), and pelvic radiotherapy, followed by total mesorectal excision surgery. He was diagnosed with metastatic lung cancer 5 years later, for which he underwent a right lower lobectomy and 6-course management of chemotherapeutics with oxali and etoposide. Further, no family history of cancer was reported. After 5 years of lung lobectomy (figure 2), the patient was referred to with pruritus about 1 month before that gradually developed over the past week.

**Figure 3:**
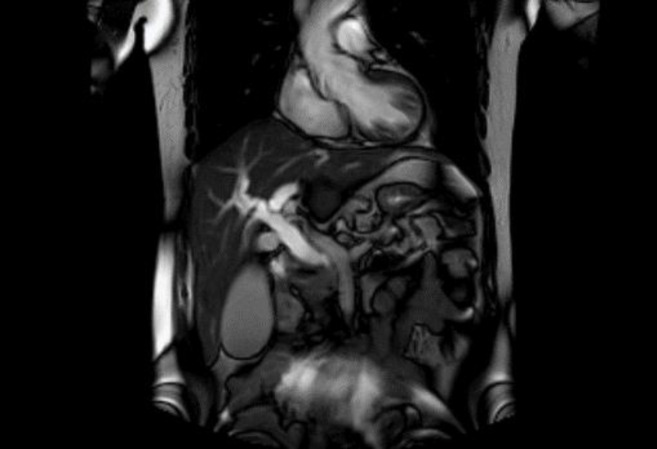
MRCP and dilated intrahepatic bile duct

**Figure 4:**
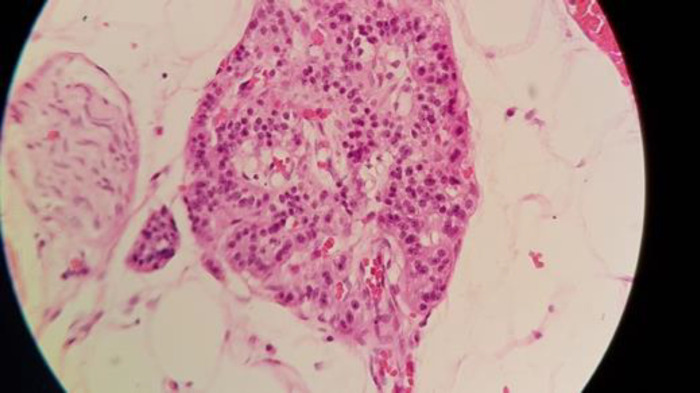
Neuroendocrin tumor

The physical examination was unremarkable; however, there was jaundice. The laboratory dataset revealed high direct bilirubin and liver function indicator (including ASt, Alt, and Alk), as well as others, such as CBC, Bun, Cr, Na, K, Bs, and Tg. Cholesterol levels were normal. Magnetic resonance cholangiopancreatography (figure 3) demonstrated a common bile duct filling defect with morphology related to external compression by the lesion (4). Suspecting a periampullary tumor, the patient underwent endoscopic ultrasound and retrograde cholangiopancreatography (ERCP), which indicated severe stenosis of the common bile duct and its proximal dilation, intrahepatic bile duct, and main pancreatic duct, as well as a 16-mm hypoechoic lesion. Following ERCP and FNA biopsy, a plastic stent was inserted, and the bilirubin was reduced. The patient underwent Whipple surgery (figure 4). All biochemistry elements, including bilirubin and hepatic enzymes, became normal ten days after surgery. He received FOLFOX for 4 courses accompanied by chemotherapy with 5florouracil and l-leucovorin and radiotherapy. The morphologic and immunohistochemical results indicated a well-differentiated neuroendocrine tumor (2).

## Discussion

Neuroendocrine tumors are rare; however, their prevalence has increased over the last two decades because of greater awareness and better diagnostic techniques, with around 5.25 per 100,000 people (6). Colorectal cancer is the third highest cause of cancer-related deaths worldwide, and its prevalence is gradually growing in developing countries (7). 

Teufel et al. demonstrated that chemotherapy did not significantly raise the incidence of subsequent second cancer in patients treated with additive chemotherapy compared to those untreated. Surprisingly, they showed that those with past additional treatment of FOLFOX had a lower risk of developing second cancer than those with FUFOL treatment (8). Thus, it seems that prior therapies may not have affected recent diseases.

The overall survival of patients with advanced colorectal carcinoma has improved; however, one of the long-term results in colorectal cancer survivors is the higher risk of second primary malignancies, which has gradually become more vital in clinical practice (9). Young age and earlier stages of malignancy are crucial characteristics that can improve outcomes, such as complete therapy and a normal life expectancy (10).

 Because the cancer was detected at a young age and in an earlier stage in our case, a normal life expectancy and positive outcome were expected, as well as late metastasis and second primary cancer in other organs.

Robertson D et al. showed the overall risk of different types of SPCs (the urinary bladder, female genital tract, kidney, thorax (lung, bronchus, and mediastinum), small intestine, stomach, thyroid, and melanoma) to be significantly higher in CRC patients compared to the general population. There was no statistically significant difference in the risk of second primary cancers of the prostate, pancreas, ovaries, esophagus, upper aerodigestive tract, liver and biliary tract, breast, cervix, uterus, and brain, as well as non-Hodgkin lymphoma, leukemia, and myeloma (11). The periampullary neuroendocrine tumor has a very low incidence and is not mentioned as a second primary cancer. According to Robertson et al., the median follow-up time was 4.2 years for SPCs (11). Our case presented the SPC after ten years of primary cancer. Neuroendocrine tumors of the extrahepatic bile ducts are sporadic, with only 200 occurrences recorded in the literature since 1961 (12). They are most commonly formed by enterochromaffin or Kulchitsky cells, with consistent pathologic features that typically correlate with the site of origin (13) and the ability to be developed in nearly any organ. The gastrointestinal tract, pancreas, and bronchopulmonary system are the most common sites for these tumors (14).

Because no serum marker is detected and no hormonal change occurs, the extrahepatic bile duct neuroendocrine tumor cannot typically be recognized before surgery. Despite technological breakthroughs and the availability of various diagnostic imaging technologies, as in our case, the diagnosis was feasible by utilizing ERCP and biopsy before surgery (15).

 Although the primary cancer neuroendocrine tumor in extrahepatic bile ducts is rare, our patient was involved with such rare cancer as a second primary cancer. Extrahepatic bile duct neuroendocrine tumors grow slowly, and surgical resection is the only curative treatment (16). In this case, a full surgical resection with free margins was performed, and the patient was followed-up. Finally, this study describes the formation of a rare case of neuroendocrine tumor following complete recovery from colorectal metastatic lung disease. Although such tumors are uncommon, they should be regarded as a second primary tumor in patients with colorectal adenocarcinoma, and the management of a second primary cancer as a first occurred cancer is important.
